# Theoretical Studies of Photophysical Properties of D−π−A−π−D-Type Diketopyrrolopyrrole-Based Molecules for Organic Light-Emitting Diodes and Organic Solar Cells

**DOI:** 10.3390/molecules25030667

**Published:** 2020-02-04

**Authors:** Ruifa Jin, Xiaofei Zhang, Wenmin Xiao

**Affiliations:** College of Chemistry and Life Science, Inner Mongolia Key Laboratory of Photoelectric Functional Materials, Chifeng University, Chifeng 024000, China; skyinthefifthday@126.com (X.Z.); xiaowenmin6868@163.com (W.X.)

**Keywords:** diketopyrrolopyrrole(DPP)-based molecules, photophysical properties, Charge transporting property, organic light-emitting diodes(OLEDs), organic solar cells(OSCs)

## Abstract

A series of D–π–A diketopyrrolopyrrole(DPP)-based small molecules were designed for organic light-emitting diode(OLEDs) and organic solar cell(OSCs) applications. Applying the PBE0/6-31G(d,p) method, the ground state geometry and relevant electronic properties were investigated. The first excited singlet state geometry and the absorption and fluorescent spectra were simulated at the TD-PBE0/6-31G(d,p) level. The calculated results revealed that the photophysical properties were affected through the introduction of different end groups. Furthermore, the electronic transitions corresponding to absorption and emission exhibited an intramolecular charge transfer feature. Our results suggest that the designed molecules acted not only as luminescent for OLEDs, but also as donor materials in OSCs. Moreover, they can also be used as potential electron transfer materials for OLEDs and OSCs.

## 1. Introduction

Organic semiconductors have attracted considerable interest in recent years due to their advantages over their inorganic counterparts, such as low-cost, lightweight, and flexible electronic devices [1,2,3,4,5]. In particular, small-molecule-based organic semiconductors are expected to open new possibilities in terms of optoelectronic applications in organic electronic devices including organic light-emitting diodes (OLEDs), organic solar cells (OSCs), and field effect transistors (FETs). Small-molecule-based organic semiconductors exhibit strong absorption and emission, high fluorescence quantum yields, and good charge carrier mobility [6]. Nevertheless, the lower efficiency of OLEDs and OSCs has seriously restricted their commercialization. The development of new small molecular materials with highly desirable properties remains a major challenge. Therefore, it is critically important to design and synthesize efficient multifunctional materials. These materials can serve as efficient light emitters in OLEDs, donor material for OSCs, and, simultaneously, charge transport materials [7,8]. In order to achieve high performance and enhance the power conversion efficiency (PCE) of OSCs, the frontier molecular orbitals (FMOs) energy levels of donors should match the typical acceptors. A deep HOMO (highest occupied molecular orbital) energy provides a high open circuit voltage (*V*_oc_). A relatively high LUMO (lowest unoccupied molecular orbital) energy ensures efficient charge separation. [9,10] Additionally, a lower HOMO–LUMO gap (*E*_g_)and strong absorption are required for effective harvesting of the solar photons. High charge-carrier mobility is also demanded for fast charge-carrier transport to maximize the short-circuit current (*J*_sc_). Furthermore, another key parameter is the downhill energetic driving force (Δ*E*_L-L_), which is strongly related to the efficient charge transfer. The Δ*E*_L-L_ can be estimated by the energy difference between the LUMO of donor and acceptor, which should amount to at least 0.3 eV [11,12]. Normally, the fullerene derivatives PC_61_BM ([6,6]-phenyl-C_61_-butyric acid methyl ester), bisPC_61_BM, and PC_71_BM are employed as acceptors in OSCs [13,14]. Nowadays, one of the most efficient strategies for optoelectronic materials is to design and synthesize donor–acceptor molecular systems containing a π-bridged(D–π–A) framework [15,16,17,18]. The electronic energy levels, absorption and emission spectra, intermolecular stacking, and film morphology can be tuned effectively through chemical modification of the acceptor, donor, and π-bridge fragments [19,20,21]. Amongst various small molecular material building blocks, such as benzofuran [22], triphenylamine (TPA) units [23], tetraphenylethylene (TPE) [24], selenophene [25], phenanthrene [26], pyrene [27], and diketopyrrolopyrrole (DPP) derivative, DPP derivatives have attracted much interest owing to their outstanding performance in OSCs, OLEDs, and FETs [28,29,30,31]. DPP-containing materials possess promising features such as strong absorption and emission in the visible region, excellent thermal and photo-stability, large Stokes shift, and straightforward synthetic modification [32,33,34,35,36]. The DPP unit is a widely recognized electron acceptor owing to its strong electron-withdrawing nature [37,38]. In the D–π−A molecular systems containing DPP as core, the introduction of aromatic blocks at the 2,5 position of the DPP core can tune the optical properties via π–πintermolecular interactions. Furthermore, the introduction of various end-capping groups onto the aromatic blocks can further tune the molecular properties. Recently, it has been reported that some multifunctional DPP derivatives exhibit good optical properties [39].

**Scheme 1:**
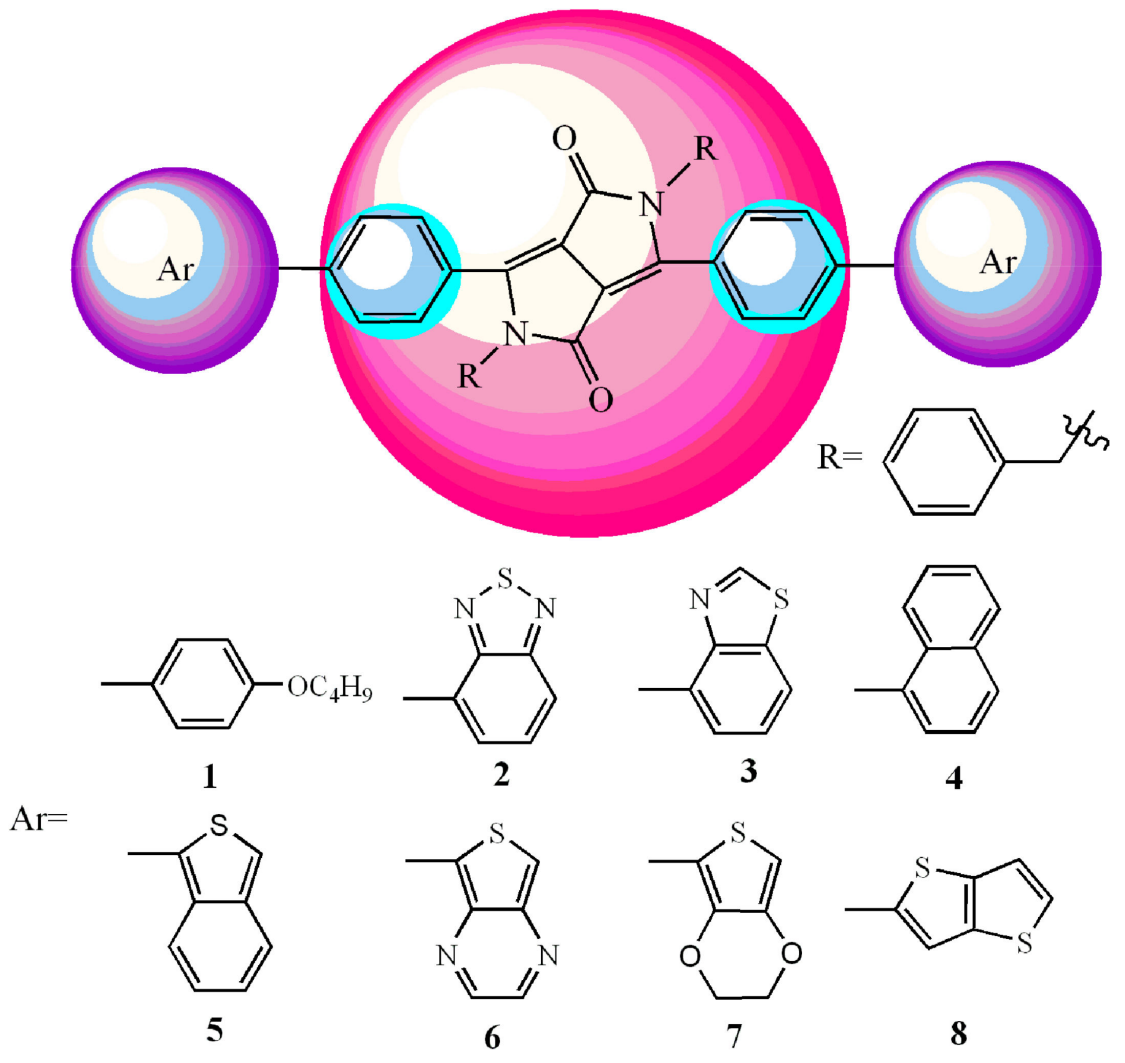
Molecular structures of the investigated molecules.

In the present work, we designed several D–π–A–π–D-structured DPP-based small molecules for OSC and OLED applications. These molecules consist of the electron deficient DPP as the core (acceptor), different planar electron-rich aromatic groups as end groups (donor), and benzene as π-bridge (Scheme 1). Via the density functional theory (DFT) and time-dependent DFT (TD-DFT) computational approach, the photophysical and charge transfer properties were systematically investigated. The FMO energies (*E*_HOMO_ and *E*_LUMO_), *E*_g_, Δ*E*_L-L_, reorganization energy (λ), and absorption and fluorescent spectra were predicted.

## 2. Results and Discussion

### 2.1. Frontier Molecular Orbitals

**Figure 1:**
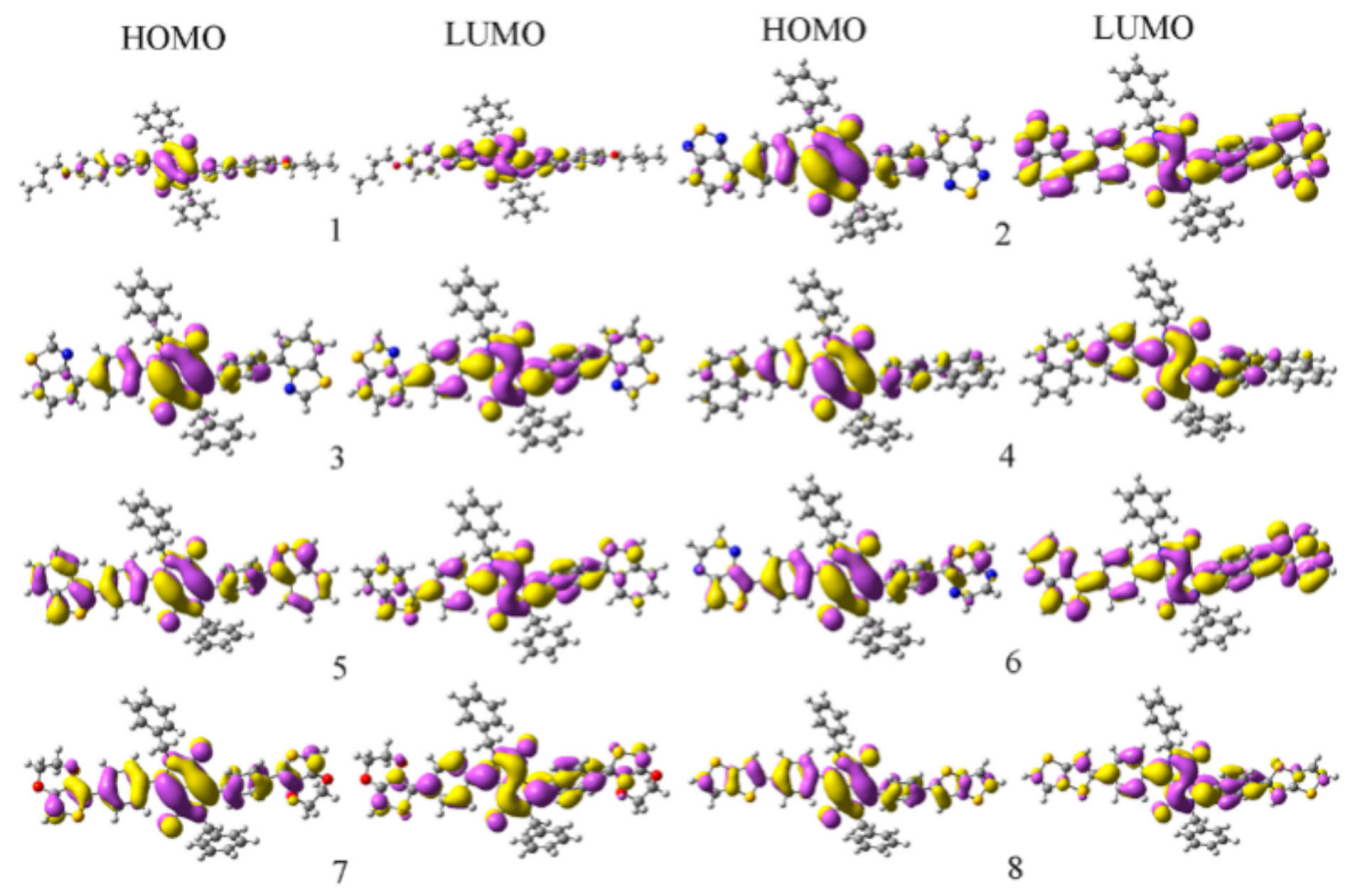
The electronic density contours of the frontier orbital for the studied compounds at the PBE0/6-31G (d,p) level.

**Figure 2:**
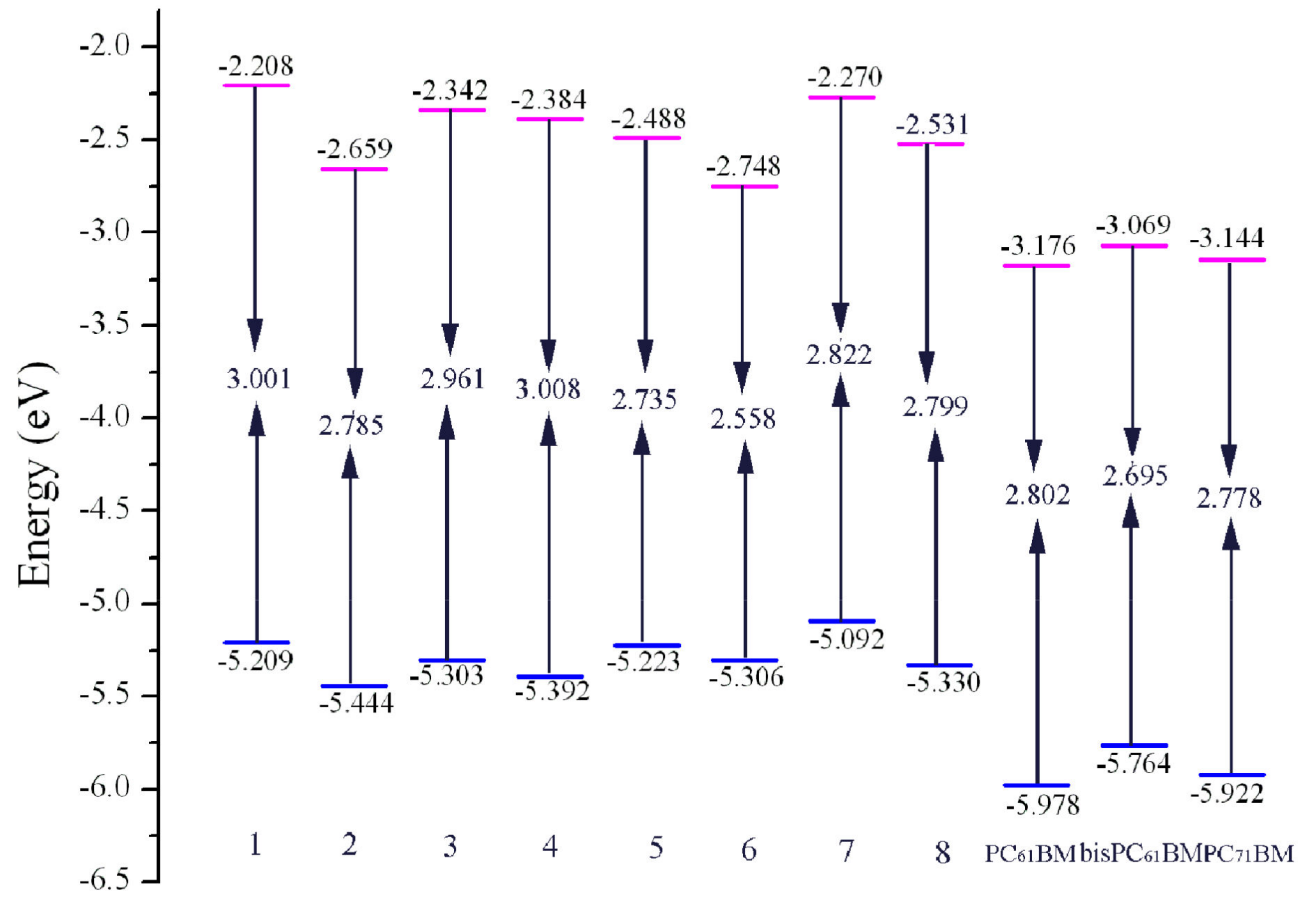
Evaluation of calculated FMO energies for investigated molecules as well as FMO energies for PC_61_BM, bisPC_61_BM, and PC_71_BM at the PBE0/6-31G (d,p) level.

To gain insight into the influence of the FMO energies on the optical and electronic properties, we examined the HOMO and LUMO contour plots of the designed molecules, as shown in Figure 1. On the basis of Mulliken population analysis, we also investigated the distribution patterns of FMOs using percentage contributions from DPP, π-bridge(BB), and end group(EG) moieties by means of partial density of states (PDOS) (see Table 1). The *E*_HOMO_, *E*_LUMO_, and *E*_g_ of **1**–**8** are plotted in Figure 2. Obviously, the FMOs exhibited π-orbital and strong delocalization features for **1**–**8**, as shown in Figure 2. It was quite obvious that, comparing the contributions of DPP, BB, and EG fragments to the LUMOs with HOMOs, the DPP fragment contributions to LUMOs were smaller than those to HOMOs for **1**–**4** and **6**–**8**, respectively. In contrast, the contribution of DPP fragment to LUMO was larger than that to HOMO for **5**.For the contributions of BB fragments, the contributions to LUMOs were larger than those to HOMOs for **1**–**5**, **7**, and **8**, respectively. The contribution to LUMO was smaller than that to HOMO for **6**. For EG fragments, the contributions to LUMOs were larger those to HOMOs for **2**, **3**, and **6** respectively. However, the contributions to LUMOs were smaller than those to HOMOs for **1**, **4**, **5**, **7**, and **8**, respectively.

Inspection of the results displayed in Table 1 revealed clearly that the excitation of the electron from the HOMOs to LUMOs led the electronic density to flow mainly from the **DPP** and **AR** fragments to **CB** fragments for **1**, **4**, **7** and **8**.For **2**, **3**, and **6**, the electronic densities mainly flowed from **DPP** fragments to **AR** and **CB** fragments. However, the electronic density mainly flowed from the **AR** fragments to **DPP** and **CB** fragment for **5**. This suggests that the different **AR** groups had obvious effects on the distribution of FMOs for the designed compounds. The percentages of charge transfer from **DPP** and **AR** fragments to **CB** fragments were 9.3%, 11.2%, 7.8%, and 10.1%for **1**, **4**, **7**, and **8**, respectively. For **2**, **3**, and **6**, the percentages of charge transfer from **DPP** fragments to **AR** and **CB** fragments were 41.4%, 15.3%, and 35.6%, respectively. The percentage of charge transfer from **AR** to **DPP** and **BB** fragment for **5** was 35.6%. Therefore, the order of the electron-donor ability of end groups for the studied compounds was benzo[c]thiophene (**5**) > thieno [3,2-b]thiophene (**8**) > 2,3-dihydrothieno[3,4-b][1,4]dioxine (**7**) > naphthalene (**4**) > butoxybenzene (**1**) > benzo[d]thiazole (**3**) > benzo[c][1,2,5]thiadiazole (**2**) > thieno[3,4-b]pyrazine (**6**). These results revealed that the **DPP** and **AR** fragments served as donors and **CB** fragments served as acceptors for **1**, **4**, **7**, and **8**. The **DPP** fragments served as donors and the **AR** and **CB** fragments served as acceptors for **2**, **3**, and **6**. For **5**, the **DPP** and **CB** fragments served as acceptors and **AR** fragment served as donors. Clearly, the vertical S_0_→S_1_ transitions for the current system possessed an intramolecular charge transfer (ICT) nature. The end groups affected the distributions of FMOs for the D–π–A–π–D molecules.

From the results displayed in Figure 2, it can be seen that the trends of *E*_HOMO_ and *E*_LUMO_ were **7** > **1** > **5** > **3** > **6** > **8** > **4** > **2** and **1** > **7** > **3** > **4** > **5** > **8** > **2** > **6**, respectively. This suggests that molecules **2**–**6** and **8** were able to decrease the *E*_HOMO_ and *E*_LUMO_ compared with molecule **1**. However, molecule **7** could increase/decrease the *E*_HOMO_/*E*_LUMO_ compared with that of molecule **1**. Furthermore, the predicted *E*_g_ sequence was **4** > **1** > **3** > **7** > **8** > **2** > **5** > **6**. Obviously, molecules **2**, **3**, and **5**–**8** could narrow, whereas molecule **4** could decrease the *E*_g_ compared with that of molecule **1**. Obviously, the introduction of benzo[c][1,2,5]thiadiazole (**2**), benzo[d]thiazole (**3**), benzo[c]thiophene (**5**), thieno[3,4-b]pyrazine (**6**), 2,3-dihydrothieno[3,4-b][1,4]dioxine (**7**), and thieno[3,2-b]thiophene (**8**) end groups led to narrower *E*_g_ values compared to molecules with a butoxybenzene (**1**)end group. However, the introduction of a naphthalene (**4**) end group decreased the *E*_g_ compared with that of molecule **1**. This implied that the introduction of different **AR** fragments (donor groups) to the DPP led to the change of the *E*_HOMO_, *E*_LUMO_, and *E*_g_ values for its derivatives. These results indicated that these D–π–A molecules can lower the band gap and extend the absorption spectrum towards longer wavelengths. The absorption and fluorescence spectra can be tuned by donor groups. Consequently, the designed molecules except for **4** may possess longer absorption and fluorescence wavelengths compared with those of molecule **1**. Therefore, it can be concluded that the *E*_HOMO_, *E*_LUMO_, and *E*_g_ of the designed D–π–A–π–D molecules can be tuned via different end groups.

### 2.2. Match between Donor and Acceptor Material

It is worth noting that the match between donor and acceptor is crucial for OSC devices. Namely, donor materials should possess suitable FMO energy levels. Firstly, with the aim of efficient electron transfer, the *E*_LUMO_ of the donor should be higher than that of the acceptor. Additionally, the Δ*E*_L-L_ should be larger than the binding energy (0.2−1.0 eV) [40,41], and should reach at least 0.3 eV. Secondly, in order to improve the performance of OSCs, donor materials should exhibit higher *J*_sc_ and *V*_oc_ values and efficient charge transfer. Therefore, lower *E*_g_s are required for ensuring the successful harvesting of sunlight and to enhance the *J*_sc_. A large difference between the *E*_HOMO_ of the donor and the *E*_LUMO_ of the acceptor is favorable for enhancing the *V*_oc_ and efficient exciton dissociation [42,43,44,45].

We took PC_61_BM, bisPC_61_BM, and PC_7__1_BM as acceptors for the current system (see Figure 2). We calculated the Δ*E*_L-L_ of **1**–**8** (see Table 2). As visualized in Figure 2, the *E*_LUMO_s of **1**–**8** were positioned above those of PC_61_BM, bisPC_61_BM, and PC_7__1_BM, respectively. When PC_61_BM, bisPC_61_BM, and PC_7__1_BM were taken as acceptors, the predicted Δ*E*_L-L_ of **1**–**8** was 0.428–0.968, 0.321–0.861, and 0.396–0.963 eV, respectively. Obviously, they are all exceeded 0.3 eV. As a consequence, the electron transfer to acceptors was efficient for these molecules. On the other hand, the *E*_HOMO_ of the designed molecules was lower by 1.916, 2.023, and 1.9481 eV than the *E*_LUMO_ of PC_61_BM, bisPC_61_BM, and PC_7__1_BM, respectively. With the above considerations, the designed molecules possess suitable FMO energies to match those of the three typical fullerene acceptors. Therefore, the FMOs of these molecules can be tuned via planar electron-rich aromatic end groups to match PC_61_BM, bisPC_61_BM, and PC_7__1_BM acceptors.

### 2.3. Absorption and Fluorescent Properties

**Figure 3:**
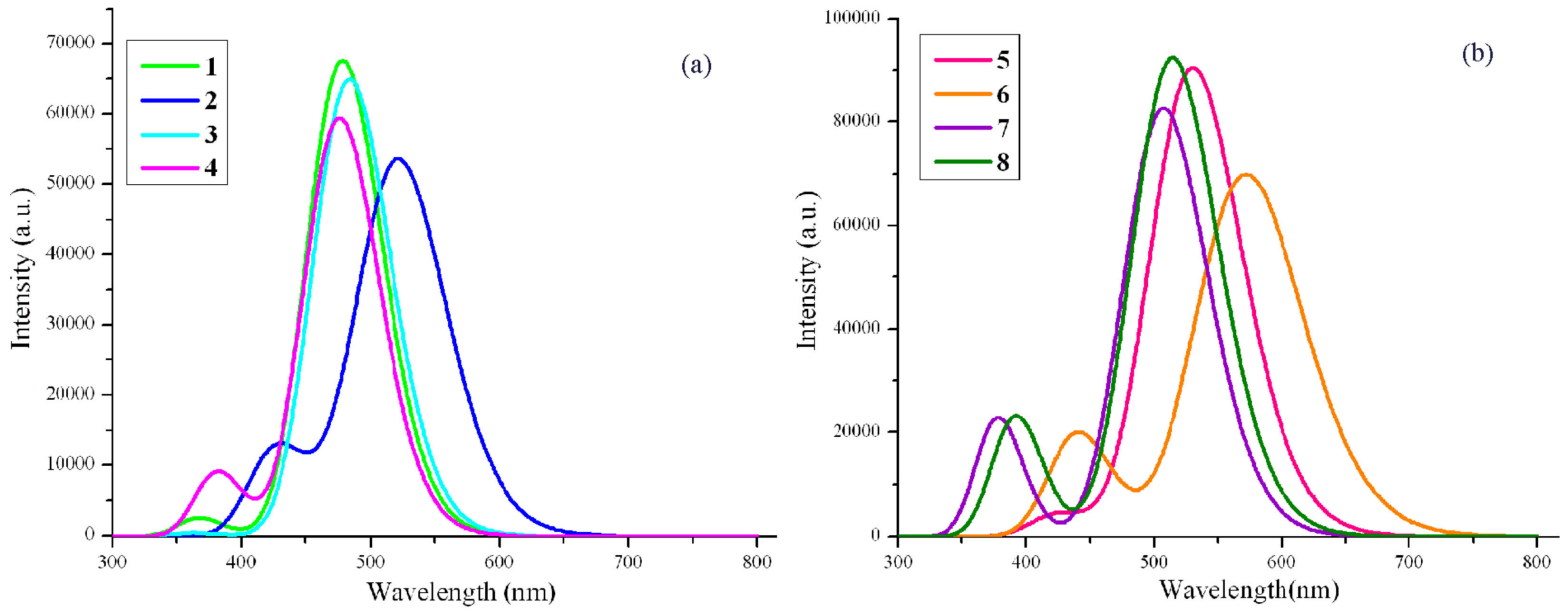
The calculated absorption spectra of the investigated molecules (value of full width at half maximum was 3000 cm^−1^). (**a**) Molecules **1**–**4**; (**b**) Molecules **5**–**8**.

**Figure 4:**
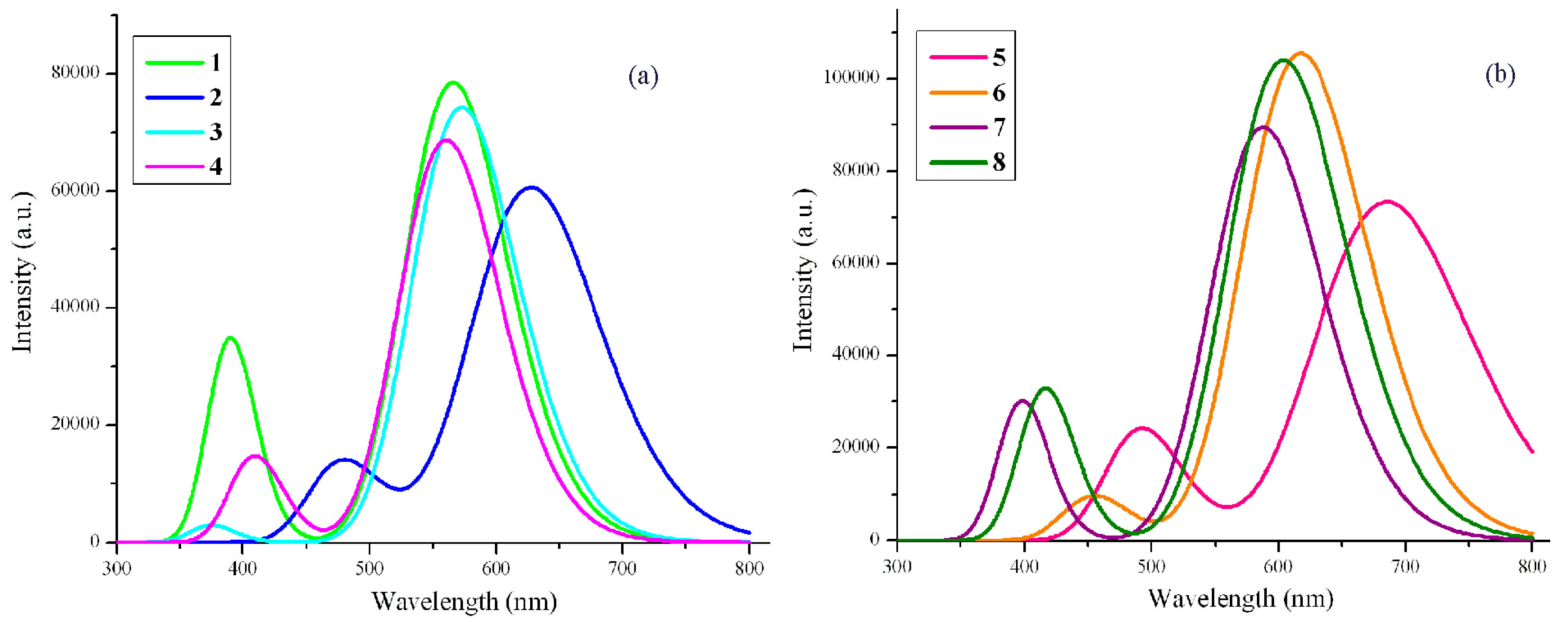
The calculated fluorescence spectra of the investigated molecules (value of full width at half maximum was 3000 cm^−1^). (**a**) Molecules **1**–**4**;(**b**) Molecules **5**–**8**.

Table 3 and Table 4 collect the predicted properties of the absorption and fluorescence spectra of the designed molecules, respectively. The simulated absorption and fluorescence spectra of **1**–**8** are shown in Figure 3 and Figure 4. For the absorption spectra, clearly, they were mainly derived from HOMO → LUMO transitions with 71% contributions for **1**–**8**. The longest wavelengths of absorption (*λ*_abs_) of molecules **2**, **3**, and **5**–**8** showed bathochromic shifts 43.4, 5.4, 52.1, 93.7, 29, and 36.1 nm, respectively, whereas molecule **4** exhibited a hypsochromic shift of 2.3 nm compared with molecule **1**. At the same time, the *λ*_abs_ was in the order of **6** > **5** > **2** > **8**> **7** >**3** > **1** > **4**, which was in excellent agreement with the corresponding reverse order of *E*_g_ values. Moreover, it was noted that molecules **6**–**8** had larger *f* value than that of **1**, while the while the corresponding *f* values of **2**–**5** were slightly less than that of **1**, respectively. Generally, a larger *f* value corresponds to a larger experimental absorption coefficient or stronger fluorescence intensity. This suggests that molecules **2**, **3**, and **5**–**8** were able to increase the *λ*_abs_ values compared with molecule **1**. On the other hand, molecule **4** did not significantly affect the *λ*_abs_ compared with molecule **1**. Therefore, the designed molecules can be used as donor materials for OSC applications.

The longest wavelengths of fluorescence (*λ*_flu_) of **1**–**8** mainly originated from the LUMO → HOMO excitations, as shown in Table 4. Similar to those absorption spectra, the *λ*_fl__u_ of molecules **2**, **3**, and **5**–**8** showed bathochromic shifts 62.3, 6.8, 119.9, 51.5, 22, and 37.9 nm compared with molecule **1**, respectively. In contrast, molecule **4** exhibited a hypsochromic shift of 5.3 nm compared with that of **1**. The *λ*_fl__u_ values were in the sequence **5** > **2** > **6** > **8** > **7** > **3** > **1** > **4**. Furthermore, the *f* values of **2**–**5** were slightly less than that of **1** and the corresponding values of molecules **6**–**8** were larger than that of molecules **1**, respectively. Therefore, the designed molecules had high fluorescent intensity. As a consequence, they can be used as luminescent materials for OLEDs, particularly for **6**–**8**.

The results displayed in Table 2 and Table 3 revealed that the absorption and fluorescence spectra of the designed molecules could be affected significantly by end groups. The designed molecules exhibited larger absorption coefficient and stronger fluorescence intensity. It suggests that these molecules could serve not only as luminescent for OLEDs, but also as donor materials in OSCs.

### 2.4. Reorganization Energies and Stabilities

The predicted *λ*_e_ and *λ*_h_ values of **1**–**8** are listed in Table 5. It was quite clear that the *λ*_h_ values of **1**–**8** were larger than those of *λ*_e_, respectively. This suggested that rates of electron transfer may have been higher than rates of hole transfer for **1**–**8**. Interestingly, molecule **1** possessed both the largest *λ*_e_ and *λ*_h_ values, indicating that the introduction of different end groups could lower the *λ*_e_ and *λ*_h_ for the designed molecules. Furthermore, molecules **5** and **6** exhibited the smallest *λ*_h_ and *λ*_e_, respectively. From these results, it can be seen that the introduction of different end groups was favorable for hole and electron transfer. They may act as electron transport materials in OLEDs and OSCs. 

Usually, the stability of materials can be predicted by means of the *η* value. As shown in Table 5, the *η* value of molecule **4** was larger than the value of molecule **1**. However, as expected, molecules **2**, **3**, and **5**–**8** possessed slightly smaller than that of molecule **1**. Compared with molecule **1**, the stabilities of **2**, **3** and **5**–**8** decreased slightly because of their steric hindrances. This suggested that the end groups had a little effect on the stability of molecules.

**Figure 5:**
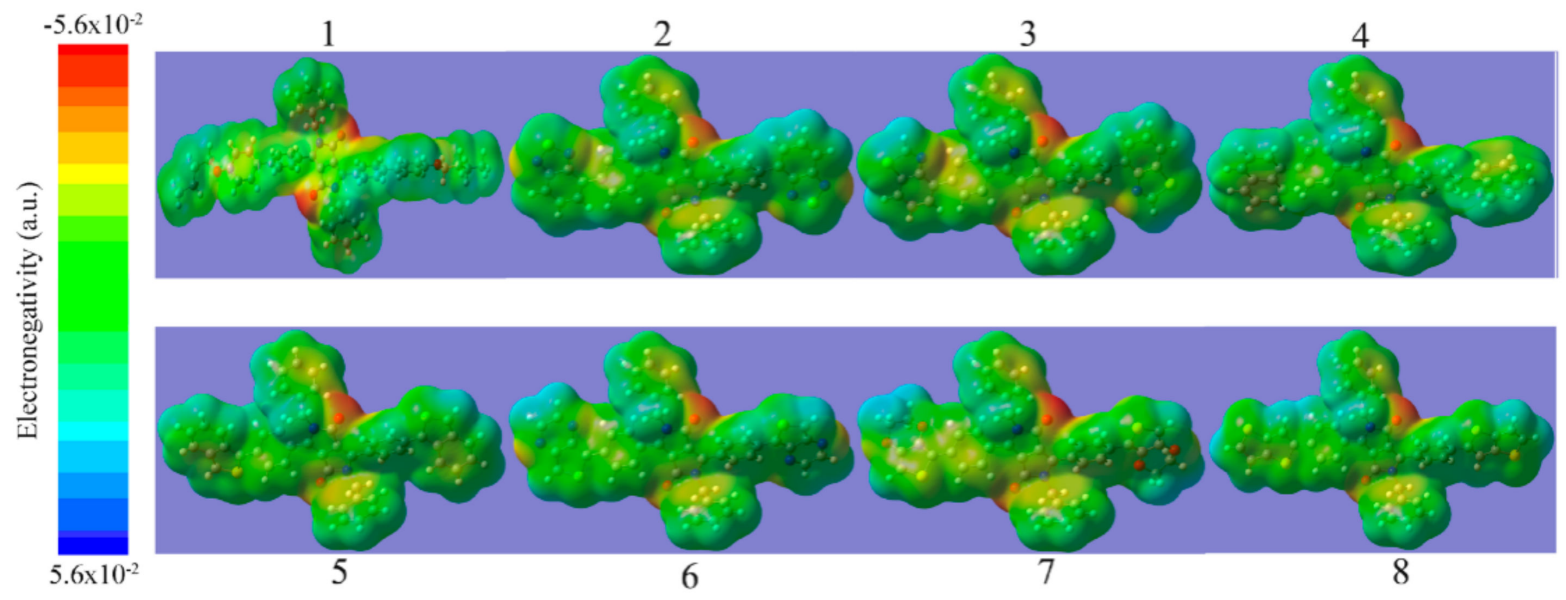
Electrostatic surface potentials for designed molecules. Regions of higher and lower electron density are shown in red and blue, respectively (values in atomic units).

Another way to evaluate the stability of material is to analyze their electrostatic surface potentials. Therefore, the electrostatic surface potentials of the designed molecules were calculated and are plotted in Figure 5. The high negative charges of **1**–**8** resided at the two oxygen atoms of DPP moieties, as visualized in Figure 5. The reason for this may be the presence lone pairs on oxygen atoms. In contrast, partial positive charges were found on the aromatic end groups. It was observed that molecules **1**–**8** had similar positive and negative potential distributions, implying that they possessed the same magnitude of photostability. Apparently, these results also revealed that the introduction of different end groups lightly affected on the stability of the molecules.

## 3. Materials and Methods

### Computational Methods

**Figure 6:**
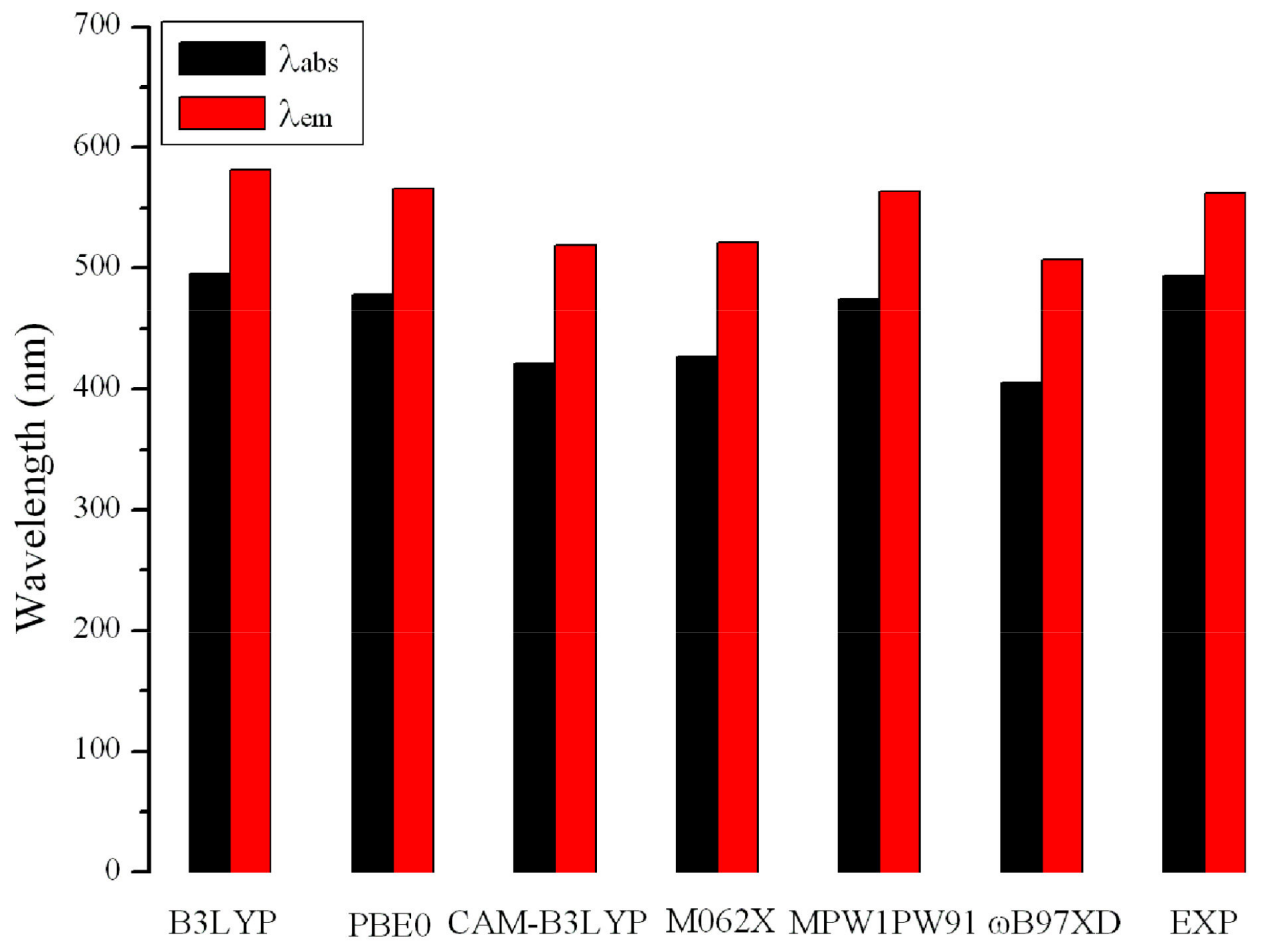
Calculated absorption and fluorescence wavelengths (*λ*_abs_ and *λ*_flu_) of molecule **1** using various functionals, together with the experimental result.

All the calculations were carried out using the Gaussian 09 suite of programs [46]. The DFT was employed to perform the geometry optimization and frequency calculations of the molecules in ground states (S_0_). The frequency analysis characterized that the optimized structures are true minima. The equilibrium geometries of the molecules in the first excited singlet state (S_1_) were optimized by mean of TD-DFT method. On the basis of the optimized structures in S_0_ and S_1_, the absorption and fluorescent spectra were simulated by TD-DFT method, respectively. With the aim of choosing a choose reasonable method, different functionals were taken to optimize the geometry of **1** in S_0_ and S_1_. These functionals contained, for example, B3LYP [47], PBE0 [48], CAM-B3LYP [49], M062X [50], MPW1PW91 [51], and *ω*B97XD [52]. Under the optimized structures in S_0_ and S_1_, the absorption and fluorescent spectra of molecule **1** were predicted using the TD-DFT method. The *λ*_abs_ and *λ*_flu_ values are plotted in Figure 6. The tested results revealed that the *λ*_abs_ and *λ*_flu_ (478.2 and 565.6 nm) using the PBE0 (583 nm) method were well able to reproduce the experimental results (494 and 562 nm) [39], and the deviations were 15.8 and 3.6 nm, respectively. The Stokes shift was 87.4 nm, which was comparable to the experimental 68 nm. As a consequence, the PBE0 method was the best choice with which to investigate our system. The PBE0 method was also used to optimize the acceptors PC_61_BM, bisPC_61_BM, and PC_71_BM. The 6-31G (d,p) basis set was used for all calculations.

It is commonly known that reorganization energy (*λ*) is a key parameter for charge transfer rates [53,54]. Lower electron (*λ*_e_) and hole (*λ*_h_) reorganization energies are beneficial for the higher electron and hole transfer rates, respectively. In this work, we only considered the internal *λ*, ignoring any environmental relaxation and changes. The *λ*_e_ and *λ*_h_ values were predicted at the PBE0/6-31G (d,p) level on the basis of the single-point energy. The *λ*_e_ and *λ*_h_ values were evaluated via the following equations [55]:

(1) $$\lambda e=\left(E_{0}^{-}-E_{-}^{-}\right)+\left(E_{-}^{0}-E_{0}^{0}\right)$$

(2) $$\lambda h=\left(E_{0}^{+}-E_{+}^{+}\right)+\left(E_{+}^{0}-E_{0}^{0}\right)$$

where $E_{0}^{\pm }$ is the energy of cation/anion structure based on the optimized neutral structure. Conversely, $E_{\pm }^{0}$ represents the energy of the neutral structure based on optimized cation/anion structure. Similarly, $E_{\pm }^{\pm }$ is the energy of cation/anion structure based on the optimized cation/anion structure, while $E_{0}^{0}$ is the energy of the neutral molecule at ground state.

It was critically important to evaluate the stability of the material in OSC and OLED devices. The absolute hardness (*η*) of materials can be used as useful criterion with which to investigate the stability of the material. The *η* values can be predicted using the following equation [56,57]:

(3) $$\eta =\frac{1}{2}\left(\frac{\partial \mu }{\partial N}\right)=\frac{1}{2}\left(\frac{\partial^{2}E}{\partial N^{2}}\right)=\frac{AIP-AEA_{}}{2}$$

where *μ* and *N* are the chemical potential and total electron number, respectively. *AIP* and *AEA* correspond to the adiabatic ionization potential and adiabatic electron affinity, respectively.

The *AIP* is the energy difference between the cation radical and its neutral species, while the *AEA* represents the energy difference between the neutral and its anion radical molecules. The PBE0/6-31G (d,p) method was applied to calculate the *AIP* and *AEA* values of the molecules. The electrostatic surface potentials can also be used to estimate the stability properties of molecules [58,59,60]. Therefore, we calculated the electrostatic surface potentials of molecules at the PBE0/6-31G (d,p) level.

## 4. Conclusions

Several D–π–A-type DPP-based small molecules were designed for OLED and OSC applications. Their photophysical and charge transfer properties were investigated using DFT and TD-DFT computational approaches. The calculated results revealed that the photophysical properties were affected through the introduction of different end groups. Furthermore, the electronic transitions corresponding to absorption and emission exhibited an intramolecular charge transfer feature. Additionally, the designed molecules possessed suitable FMO energies to match those of three typical fullerene acceptors, PC_61_BM, bisPC_61_BM, and PC_7__1_BM. It was disclosed that the designed molecules acted not only as luminescent for OLEDs, but also as donor materials in OSCs. Moreover, they could also be used as potential electron transfer materials for OLEDs and OSCs.

## Figures and Tables

**Table 1:** The highest occupied molecular orbit (HOMO) and lowest unoccupied molecular orbit (LUMO) contributions of individual fragments (in%) to the frontier molecular orbitals (FMOs) of **1**–**8** at the PBE0/6-31G (d,p) level.

Species	HOMOs			LUMOs		
	DPP ^a^	CB ^b^	Ar ^c^	DPP	CB	Ar
**1**	71.0	19.2	9.8	66.1	28.5	5.4
**2**	75.3	19.0	5.7	33.9	21.1	45.0
**3**	75.5	19.4	5.1	60.2	30.3	9.5
**4**	73.1	19.0	7.9	64.3	30.2	5.6
**5**	47.3	19.7	33.0	54.8	29.8	15.5
**6**	63.9	21.3	14.9	28.3	21.1	50.6
**7**	61.2	22.6	16.3	58.2	30.4	11.5
**8**	61.3	21.0	17.7	56.3	31.1	12.5

^a^ DPP: diketopyrrolopyrrole fragment; ^b^ CB: conjugate bridge moieties; ^c^ Ar: aromatic groups.

**Table 2:** The calculated Δ*E*_L-L_ values for **1**–**8** at the PBE0/6-31G (d,p).

Species	Δ*E*_L-L_^a^	Δ*E*_L-L_^b^	Δ*E*_L-L_^c^
**1**	0.968	0.861	0.936
**2**	0.517	0.410	0.485
**3**	0.834	0.727	0.802
**4**	0.792	0.685	0.760
**5**	0.688	0.581	0.656
**6**	0.428	0.321	0.396
**7**	0.906	0.799	0.874
**8**	0.645	0.538	0.613

^a^ Δ*E*_L-L_ for PC_61_BM as acceptor; ^b^ Δ*E*_L-L_ for bisPC_61_BM as acceptor; ^c^ Δ*E*_L-L_ for PC_7__1_BM as acceptor.

**Table 3:** Predicted longest wavelengths of absorption, corresponding oscillator strength *f*, and main assignment of **1**–**8** at the TD- PBE0/6-31G (d,p)//PBE0/6-31G (d,p) level.

Species	λ_ab__s_ (nm)	*f*	Assignment
**1**	478.2	0.93	HOMO → LUMO (0.71)
**2**	521.6	0.74	HOMO → LUMO (0.70)
**3**	483.6	0.90	HOMO → LUMO(0.71)
**4**	475.9	0.82	HOMO → LUMO(0.71)
**5**	530.3	1.25	HOMO → LUMO (0.71)
**6**	571.9	0.96	HOMO → LUMO(0.70)
**7**	507.2	1.14	HOMO → LUMO(0.71)
**8**	514.3	1.28	HOMO → LUMO(0.70)
Exp ^a^	494		

^a^ Experimental data for **1** were taken from Reference [39].

**Table 4:** Predicted longest wavelengths of fluorescence, corresponding oscillator strength *f*, and main assignment of **1**–**8** at the TD-PBE0/6-31G (d,p)//TD-PBE0/6-31G (d,p) level.

Species	λ_flu_	*f*	Assignment
**1**	565.6	1.08	HOMO ← LUMO (0.71)
**2**	627.9	0.83	HOMO ← LUMO (0.71)
**3**	572.4	1.03	HOMO ← LUMO(0.71)
**4**	560.3	0.95	HOMO ← LUMO(0.71)
**5**	685.5	1.01	HOMO ← LUMO (0.71)
**6**	617.1	1.46	HOMO ← LUMO(0.71)
**7**	587.6	1.23	HOMO ← LUMO(0.71)
**8**	603.5	1.44	HOMO ← LUMO(0.71)
Exp ^a^	562		

^a^ Experimental data for **1** were taken from Reference [39].

**Table 5:** Calculated λ_e_, λ_h_, and *η* values (all in eV) of **1**–**8** at the PBE0/6-31G (d,p) level.

Species	*λ*_h_	*λ*_e_	*η*
**1**	0.401	0.343	2.284
**2**	0.374	0.240	2.174
**3**	0.386	0.322	2.277
**4**	0.364	0.315	2.305
**5**	0.325	0.260	2.104
**6**	0.356	0.222	2.017
**7**	0.392	0.278	2.180
**8**	0.380	0.263	2.145

## Abbreviations

OSCs	Organic solar cells
OLEDs	Organic light-emitting diodes
FETs	Field effect transistors
Δ*E*_L-L_	Downhill energetic driving force
DPP	Diketopyrrolopyrrole
ICT	Intramolecular charge transfer
DFT	Density function theory
TD-DFT	Time dependent density function theory
FMOs	Frontier molecular orbital energies
HOMO	Highest occupied molecular orbital
LUMO	Lowest unoccupied molecular orbital
